# An exploration of the sociodemographic and health conditions associated with self-rated wellbeing for Aboriginal and Torres Strait Islander adults

**DOI:** 10.1186/s13104-021-05794-3

**Published:** 2021-10-02

**Authors:** A. Gall, A. Diaz, G. Garvey, K. Anderson, D. Lindsay, K. Howard

**Affiliations:** 1 Wellbeing and Preventable Chronic Disease Division, Menzies School of Health Research, Charles Darwin University, Casuarina, NT Australia; 2 School of Public Health, Faculty of Medicine & Health, University of Sydney, Sydney, NSW 2006 Australia; 3 School of Public Health, University of Queensland, Brisbane, Australia; 4 Menzies Centre for Health Policy and Economics, Faculty of Medicine and Health, University of Sydney, Sydney, NSW 2006 Australia

**Keywords:** Wellbeing, Well-being, Quality of life, Indigenous peoples, Visual analogue scale, Australia, Aboriginal and Torres Strait Islander people, Sociodemographic, Mental health, Comorbidities

## Abstract

**Objective:**

To identify sociodemographic factors and health conditions associated with self-rated wellbeing for Aboriginal and Torres Strait Islander adults. Participants were recruited via investigator networks and an online panel provider with an established nationwide panel of Aboriginal and Torres Strait Islander adults. Those interested were invited to complete a survey that included an assessment of wellbeing using a visual analogue scale. Data was collected from October–November 2019 and August–September 2020. Exploratory analyses were conducted to ascertain factors associated with self-rated wellbeing for Aboriginal and Torres Strait Islander adults.

**Results:**

Having more than enough money to last until next pay day, full-time employment, completion of grade 12, having a partner, and living with others were significantly associated with higher wellbeing among Aboriginal and Torres Strait Islander adults. A self-reported history of depression, anxiety, other mental health conditions, heart disease, or disability were associated with lower self-rated wellbeing scores. Our findings indicate a need for further investigation among these socioeconomic and patient groups to identify how to improve and support the wellbeing of Aboriginal and Torres Strait Islander adults.

**Supplementary Information:**

The online version contains supplementary material available at 10.1186/s13104-021-05794-3.

## Introduction

There has been increased attention on understanding, defining and measuring wellbeing for populations worldwide, including the perspectives and considerations of cultural groups [1–3]. Understanding wellbeing from an Aboriginal and Torres Strait Islander perspective (herein respectfully referred to as Indigenous Australians) has received recent attention due to the imperative for Australian governments to improve Indigenous Australians’ health and wellbeing. Indigenous Australians understanding of wellbeing is holistic and includes physical, emotional, spiritual, cultural, and socio-political aspects of life of the individual and their community [4–6]. While no nationally relevant measure of wellbeing for Indigenous Australians is currently available [7], gaining an understanding of groups who are at risk of poor wellbeing is clinically pertinent. Such information may inform the provision of targeted programs and services to better support the wellbeing of Indigenous Australians.

The current study, part of the larger *What Matters 2Adults Study *[7], aims to identify sociodemographic factors and health conditions associated with self-rated wellbeing for Indigenous Australian adults.

## Main Text

### Methods

This exploratory study recruited Indigenous Australian adults (≥ 18 years) via investigator networks and an online panel provider (Dynata) with an established nationwide panel of Indigenous Australian adults. Potential participants received study information and those interested completed an online consent form before completing the online survey. Survey data was collected during October–November 2019 (n = 309) and August–September 2020 (n = 354). There were 42 respondents who completed the survey at both times. A sensitivity analysis was conducted removing the data collected from the first round of the survey for the 42 participants who completed the survey at both rounds. The direction and size of between-group differences in self-rated wellbeing scores did not materially change with the exclusion of these data. As such, all 663 responses were treated as independent observations of wellbeing.

### Data collection, measures and analysis

Participants were asked to self-rate their *“…overall wellbeing at the moment”* using a 100-point visual analogue scale, with zero indicating the worst wellbeing they could imagine and 100 indicating the best wellbeing they could imagine (see Additional file 1). Self-reported sociodemographic, socioeconomic and health conditions were collected, including: age, gender, Indigenous status, main language spoken at home, residential area, relationship status, household size, highest level of education, employment status, financial situation and comorbid conditions (see Table 1). Composite variables were created to measure the number of physical comorbidities, and the presence/absence of any mental health comorbidity.

**Table 1:** Participant characteristics and unadjusted self-rated wellbeing score (n = 663)

Characteristic	n (%)	Wellbeing score	
		Mean (SD)	p-value^a^
Total	663 (100%)	66.1 (24.5)	n/a
Age in years, median (IQR)	45 (35–56)	n/a	n/a
Age group			
18–29 years	112 (16.9)	64.2 (22.8)	< 0.001
30–44 years	246 (37.1)	70.9 (23.0)	
45–59 years	178 (26.8)	59.7 (26.3)	
60 + years	127 (19.2)	67.5 (25.6)	
Gender^b^			
Male	271 (40.9)	67.9 (25.3)	0.08
Female	389 (58.7)	65.1 (23.9)	
Indigenous status			
Aboriginal	607 (91.6)	66.5 (24.4)	0.07
Torres Strait Islander	21 (3.2)	70.5 (20.7)	
Aboriginal and Torres Strait Islander	35 (5.3)	57.3 (27.3)	
Main language spoken at home^c^			
English	604 (91.1)	65.7 (24.5)	0.22
Torres Strait Islander or Aboriginal language	58 (8.8)	70.5 (23.8)	
Residential area			
Metropolitan	332 (50.1)	64.7 (24.8)	0.12
Rural/regional	331 (49.9)	67.6 (24.1)	
Relationship status			
Partnered	407 (61.4)	70.4 (21.6)	< 0.001
Single	237 (35.8)	59.2 (27.4)	
Other	19 (2.9)	61.1 (23.8)	
Household size^d^			
Range	1 to 10	n/a	n/a
Sole occupier	126 (19.0)	56.2 (28.3)	< 0.001
2 People	167 (25.2)	67.6 (22.7)	
3 People	128 (19.3)	65.6 (24.6)	
4 People	119 (18.0)	75.1 (18.9)	
5 + People	122 (18.4)	66.2 (24.1)	
Highest level of education			
Grade 10	148 (22.3)	58.8 (26.6)	< 0.001
Grade 12	84 (12.7)	69.7 (22.3)	
TAFE certificate/diploma or trade certificate	220 (33.2)	65.3 (24.5)	
University	211 (31.8)	70.8 (22.4)	
Employment status			
Employed casual	42 (6.3)	65.1 (23.5)	< 0.001
Employed part-time	75 (11.3)	65.9 (24.1)	
Employed full-time	250 (37.7)	74.4 (19.1)	
Not working	64 (9.7)	58.5 (28.6)	
Student	27 (4.1)	63.4 (24.8)	
Retired/pension	113 (17.0)	60.3 (27.7)	
Home duties	71 (10.7)	58.1 (24.3)	
Other	21 (3.2)	55.9 (26.9)	
Financial situation			
Not enough money to last until next pay day	224 (33.8)	58.5 (24.6)	< 0.001
Just enough money to last until next pay day	288 (43.4)	67.1 (25.5)	
More than enough money to last until next pay day	151 (22.8)	75.6 (18.0)	
physical comorbidities			
Nil	165 (24.9)	71.1 (22.0)	< 0.001
1–2	228 (34.4)	70.1 (22.9)	
3–4	128 (19.3)	63.2 (24.2)	
5–6	76 (11.5)	63.2 (26.3)	
7+	66 (10.0)	49.2 (26.0)	
Mental health comorbidities			
Nil	323 (48.7)	74.5 (19.8)	< 0.001
Any	340 (51.3)	58.1 (25.8)	
Depression			
No	384 (57.9)	72.9 (20.0)	< 0.001
Yes	279 (42.1)	56.8 (26.9)	
Anxiety			
No	406 (61.2)	72.5 (20.7)	< 0.001
Yes	257 (38.8)	56.1 (26.7)	
Other mental health comorbidities			
No	583 (87.9)	69.0 (22.7)	< 0.001
Yes	80 (12.1)	45.1 (26.5)	

*IQR* interquartile range, *n* number; *SD* standard deviation

^a^Two-tailed two-sample t-test for variables with two categories and ANOVA for variables with three or more categories

^b^3 reported ‘other’; data not shown

^c^1 reported ‘other’; data not shown

^d^Excluded outlier (n = 1)

Differences in mean wellbeing scores were tested using t-tests and ANOVA. Variables not statistically significantly associated with wellbeing at univariate level (using cut-off p < 0.1) were excluded in subsequent multivariable analyses. Two multiple linear regression models were conducted to produce adjusted estimates of the differences in wellbeing scores. Model 1 included sociodemographic and socioeconomic variables and composite measures relating to comorbidities (number of physical conditions participants reported having and presence of any mental health comorbidity). Model 2 included individual comorbidities eligible for inclusion based on univariate analyses. Both models were adjusted for age and calendar period at time of survey completion. As age did not have a linear relationship with wellbeing, it was modelled as a categorical variable. There were no missing values, however, one extreme outlier in household size was excluded in the multivariable analyses. All analyses were conducted in Stata v15 [8].

### Results

A total of 663 Indigenous Australian adults (60% female), with median age of 45 years (median; IQR 35–56), completed the online survey (47% in the first round). Participants from all mainland Australian states and territories were included, with 41% of the sample from New South Wales and 2% from the Australian Capital Territory. The distribution of participants across the states and territories was broadly reflective of the distribution of the national Indigenous Australian population[9]. Participant characteristics and mean self-rated wellbeing scores are described in Table 1. Overall, the unadjusted mean wellbeing score was 66.1 (SD 24.5). There were significant differences in the unadjusted scores based on age group, relationship status, household size, highest education, employment, financial situation and comorbidities (p < 0.05).

In multivariable analyses, the adjusted mean wellbeing score among participants was 70.3 and, on average, 3.4 points higher in the second-round of the survey compared to the first (Table 2). On average, participants aged 30–44 years and 60 + years had wellbeing scores that were 5.2 (p = 0.02) and 11.0 points (p < 0.001) higher than those aged 45–59 years. Those who reported being single rated their wellbeing, on average, 4.6 points lower than those who were partnered (p = 0.03). The mean wellbeing score was 8.9 points and 7.5 points higher, on average, for those living in households of four people (p = 0.01) and five or more people (p = 0.02) compared to sole occupiers. Participants with Grade 12 completion had wellbeing 8.1 points higher, on average, than those who completed grade 10 or below (p = 0.01). Compared to those in full-time employment, those who reported not working, or having part-time or other employment, rated their wellbeing significantly lower on average (6.8, 6.2 and 10.2 points lower, p =  < 0.01, 0.04 and 0.05, respectively). Participants who indicated having more than enough money to last until their next payday had significantly higher wellbeing, on average, compared to those who did not have enough, or had just enough money (7.6, 5.3 points lower, p =  < 0.01, 0.02, respectively). Participants with seven or more physical comorbidities had wellbeing scores 8.2 points lower, on average, than those who reported having no physical comorbidities (p = 0.03), while those with fewer physical comorbidities did not have a significantly different wellbeing score than those without physical comorbidity. Participants reporting any mental health condition experienced 11.9 points lower wellbeing, on average, than those who had none (p =  < 0.001).

**Table 2:** Self-rated wellbeing score^a^ in Aboriginal and Torres Strait Islander adults (n = 662^b^), adjusted^c, d^ for patient characteristics (Model 1)

Variable	Coefficient	95% CI	p-value
Constant (overall mean score)	70.3	62.9 to 79.7	< 0.001
Age group (referent: 45–59 years)			
18–29 years	2.1	− 3.7 to 7.9	0.48
30–44 years	5.2	0.8 to 9.7	0.02
60 + years	11.0	5.5 to 16.5	< 0.001
Relationship status (referent: partnered)			
Single	− 4.6	− 8.6 to − 0.6	0.03
Other	− 7.0	− 17.2 to 3.1	0.17
Household size^b^ (referent: sole occupier)			
2 people	3.9	− 1.7 to 9.5	0.17
3 people	4.3	− 1.6 to 10.1	0.15
4 people	8.9	2.4 to 15.4	0.01
5 + people	7.5	1.2 to 13.8	0.02
Highest level of education (referent: grade 10 or below)			
Grade 12	8.1	2.0 to 14.2	0.01
TAFE/trade certificate	3.5	− 1.3 to 8.3	0.15
University	5.0	− 0.1 to 10.0	0.05
Employment status (referent: fulltime employment)			
Casual employment	− 6.3	− 13.7 to 1.1	0.10
Part-time employment	− 6.2	− 12.0 to − 0.3	0.04
Not working	− 6.8	− 11.5 to − 2.2	< 0.01
Student	− 7.5	− 16.4 to ﻿− 1.4	0.01
Other	− 10.2	− 20.2 to − 0.1	0.05
Financial situation (referent: more than enough money)			
Just enough money	− 5.3	− 9.7 to − 0.9	0.02
Not enough money	− 7.6	− 12.6 to − 2.6	< 0.01
Physical comorbidities (referent: nil physical comorbidities)			
1–2	4.2	− 0.4 to 8.9	0.07
3–4	0.2	− 5.4 to 5.7	0.96
5–6	0.7	− 6.0 to 7.4	0.84
7 +	− 8.2	− 15.5 to − 1.0	0.03
Mental health comorbidities (referent: nil mental health)			
Any mental health	− 11.9	− 15.6 to − 8.2	< 0.001

CI confidence interval

^a^Self-rated wellbeing assessed using a wellbeing visual analogue scale (VAS)

^b^Excluded outlier (n = 1)

^c^Using multiple linear regression

^d^Further adjusted for survey time (first round: Oct–Nov 2019 and second round: Aug-Sept 2020)

After mutual adjustment for comorbid conditions in model 2, having heart disease, a disability, depression, anxiety and other mental health conditions were statistically significantly associated with lower wellbeing (Table 3).

**Table 3:** Self-rated wellbeing score^a^ in Aboriginal and Torres Strait Islander adults (n = 663), adjusted^b, c^ for comorbidities (Model 2)

Variable	Coefficient	95% CI	p-value
Constant (overall mean score)	69.0	64.5 to 73.4	< 0.001
Heart disease	− 7.1	− 13.6 to − 0.6	0.03
High cholesterol	0.2	− 4.2 to 4.5	0.94
High blood pressure	1.9	− 2.4 to 6.3	0.38
Arthritis	− 2.4	− 7.2 to 2.4	0.33
Thyroid problems	− 0.8	− 6.5 to 5.0	0.79
Stomach problems	− 2.8	− 8.0 to 2.5	0.31
Hearing loss	− 3.6	− 9.0 to 1.8	0.19
Disability	− 11.3	− 17.1 to − 5.5	< 0.001
Depression	− 5.6	− 10.0 to − 1.3	0.01
Anxiety	− 7.5	− 11.9 to − 3.1	< 0.01
Other mental health	13.1	− 18.7 to − 7.5	< 0.001

^a^Self-rated wellbeing assessed using a wellbeing visual analogue scale (VAS)

^b^Using multiple linear regression

^c^Further adjusted for age group (18–29, 30–44, 45–59, 60 + years) and survey time (first round: Oct-Nov 2019 and second round: Aug-Sept 2020)

### Discussion

In a large sample of Indigenous Australian adults, we identified several socioeconomic and sociodemographic factors and health conditions associated with wellbeing. Having more than enough money to last until next pay day, full-time employment, and completion of grade 12 were all associated with higher levels of wellbeing. Other studies identifying aspects of life that are important to the wellbeing of Indigenous Australians also found that employment, education and money are needed to achieve good wellbeing [6, 10, 11]. Our results are consistent with previous studies in other populations that identified important socioeconomic impacts on wellbeing or quality-of-life, including financial stability for respondents in China, Ghana, India, South Africa, Russia and the United States [12, 13], employment in the general Australian population [14], and higher education in the United States [13]. Socioeconomic disadvantage is consistently associated with poorer health outcomes for Indigenous Australians with cancer [15], cardiovascular disease [16, 17], psychological distress [18], and liver cirrhosis [19], and have been associated with potentially preventable hospital admissions [20]. The effect of socioeconomic disadvantage on health and wellbeing is likely to explain only some of the observed health disparities for Indigenous Australians compared to non-Indigenous Australians, with other factors, including systemic racism, contributing to inequalities across socioeconomic strata [16, 18].

Family, kinships and community connections are important to the wellbeing of Indigenous peoples in Australia [6, 10, 11], and globally [21]. In this study, wellbeing was lowest among those without partners or living on their own. This was consistent with a study of Indigenous Australians with cancer where health related quality-of-life was higher for those who were married [22]. Having a partner and/or living with others helps maintain social connections, which may mitigate the impact of social determinants of poor wellbeing [23, 24]. More work is needed to understand the role of loneliness, social connection and social isolation in wellbeing for Indigenous Australians. These factors are particularly relevant during this COVID-19 era, where public health restrictions [25–27] have limited opportunities for social interaction and connection.

Indigenous Australians have higher incidence of many common chronic diseases and are more likely to be diagnosed at a younger age and living with multiple chronic conditions [28]. Half of the participants in the current study reported having at least one mental health condition and 17% had a very high burden of physical comorbidity (≥ 7 conditions). We found participants with at least one mental health condition and/or a very high burden of physical comorbidities rated their wellbeing significantly lower than those without those comorbidities. However, there did not appear to be a linear relationship between wellbeing and number of diagnosed physical conditions reported. This finding suggests the relationship between physical comorbidity and wellbeing goes beyond the number of conditions a person has. This survey did not collect information on the management or severity of comorbid conditions, nor how they impacted on daily activities, which has previously been shown to be pertinent for wellbeing and quality-of-life [29–31]. The findings may also suggest that socioeconomic and social connection factors may be more important than health state, emphasising the holistic nature of wellbeing for Indigenous peoples [32].

In the current study, depression, anxiety, other mental health conditions, heart disease, and disability were associated with wellbeing. Recent national estimates suggest 25% of Indigenous Australian females and 23% of Indigenous Australian males have a mental illness or behavioural condition, with anxiety (17%) and depression (13%) the most common conditions [33]. The relationship between mental health and wellbeing has previously been demonstrated for the general Australian population [34, 35] and other Indigenous populations worldwide [36], highlighting the need for increased investment in preventive mental health programs. Indigenous Australians have higher rates of heart disease than non-Indigenous Australians (27% and 21%, respectively) [37], and 38% of Indigenous Australians have a disability that restricts their everyday lives, at a rate of 1.8 times that of non-Indigenous Australians [33]. Heart disease is commonly associated with disability and poor quality-of-life, however the link between disability and wellbeing is poorly understood [38, 39]. Previous reports have shown a link between chronic health conditions and their impact on wellbeing long-term [33]. Further investigation among these patient groups may provide critical information about how to improve and maintain good wellbeing for Indigenous Australians.

### Conclusion

This study explored the relationship between wellbeing and downstream social determinants of health for Indigenous Australians. Our findings suggest those with a history of mental illness, heart disease, a disability, as well as those without partners, living on their own, and who have not completed high school, are in precarious or under employment, and have financial instability are at risk of poor wellbeing. The identification of these factors may offer health and social services a way to identify Indigenous Australians at risk and provide opportunities for individual intervention. Prospective studies are needed to gain deeper understanding of the dynamic and intersectional nature of the relationships between socioeconomic wellbeing, social connection, and physical and mental health for Indigenous Australians.

### Limitations

This is one of the largest studies investigating factors associated with self-rated wellbeing among a geographically diverse respondent sample of Indigenous Australian adults. However, we were limited by a self-reported single global measure of wellbeing. In studies with non-Indigenous populations the visual analogue scale was found to measure quality-of-life comparably with multi-item questionnaires [40]. However, the use of a visual analogue scale to measure overall wellbeing has not been validated with Indigenous Australians [41–43] limiting our ability to examine specific dimensions of wellbeing. This study explored downstream social determinants of wellbeing. However, we recognise the importance of midstream determinants, such as the characteristics and accessibility of health and social services that may support or hinder wellbeing of individuals and communities, and upstream determinants, such as the political, education and justice systems of society and the structural racism that is pervasive within these systems and known to be critical for wellbeing [16, 18]. While this cross-sectional study limited our ability to infer causality, findings may inform the direction of future research to understand the nature of these relationships and develop ways to improve clinical, public health and social services capabilities to support wellbeing among Indigenous Australian adults and their communities.

## Supplementary Information

**Additional file 1.** The What Matters survey.

## Data Availability

The datasets generated and analysed during the current study are not publicly available but may be available from the authors on request.
