# Supplementary material for: An exploration of the sociodemographic and health conditions associated with self-rated wellbeing for Aboriginal and Torres Strait Islander adults
Source: BMC Res Notes. 2021 Oct 2;14:386. doi: 10.1186/s13104-021-05794-3 (PMC8487334; doi:10.1186/s13104-021-05794-3)
Supplement: Supplementary file 1 — Additional file 1. The What Matters survey. [file 13104_2021_5794_MOESM1_ESM.docx]

**The What Matters Survey**

**Aboriginal and Torres Strait Islander Adult Version**

**CONSENT PAGE**

By clicking on the CONTINUE button below, I am indicating that I understand:

- What this project is about.
- That any information I provide will be confidential and it will not be shared outside of this research project,
- That I will not be identifiable in any reports or other outputs from this study.
- That participating will take between 25-30 minutes.
- That I am free to withdraw at any time.
- That I will have the option to withdraw my data should I decide not to complete the study.
- That after completing the study my data cannot be withdrawn.
- That the data collected for this study may also be used to inform the development of other related research.
- That the ownership of Aboriginal and Torres Strait Islander knowledge and cultural heritage is retained by the informant and this will be acknowledged in research findings and in the dissemination of the research.
- That I can speak with the ethics secretariat of Human Research Ethics Committee of the Northern Territory Department of Health and Menzies School of Health Research about any concerns I have about the research or the conduct of the research (phone: (08) 8946 8687 or (08) 8946 8692 or email: ethics@menzies.edu.au.

Would you like to be contacted about testing the final wellbeing survey tool that your interview has helped to create? **YES** / **NO**

If you circled “yes”, please provide contact details below.

Email:___________________________________________________Mobile:_____________________________

Postal address: _____________________________________________ State: ________ Postcode: ________

**BEFORE WE START…**

Before you start the survey, we would like to ask a couple of questions about you.

|  | | Please click on and/or type your responses below |
| --- | --- | --- |
| How old are you? | | ✍️_________ years |
| Are you… | | Male  Female  Other |
| What is your background? | | Aboriginal  Torres Strait Islander  Aboriginal & Torres Strait Islander |
| What is your highest level of education? | Year 10 or below  Year 12 (leaving)  TAFE certificate/diploma, trade certificate  University | |
| In what town/city do you usually live? | | ✍️ _____________________________ |
| What is your current postcode? | | ✍️ _____________________________ |

**The What Matters Survey**

**Aboriginal and Torres Strait Islander Adult Version**


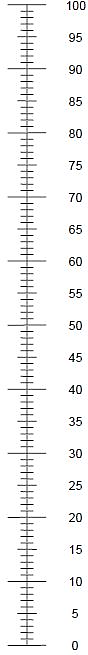


We would like to know how you would rate your overall wellbeing at the moment.

- This scale is numbered from 0 to 100.
- 0 means that your wellbeing is the worst can imagine.
- 100 means that your wellbeing is the best can imagine.
- Click the marker on the scale to indicate how you would rate your wellbeing at the moment.


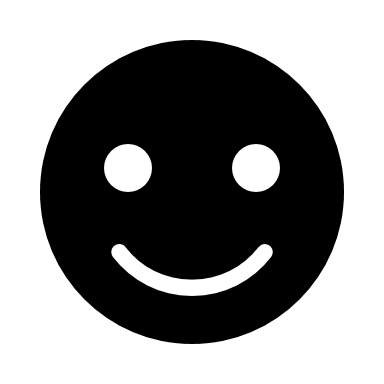

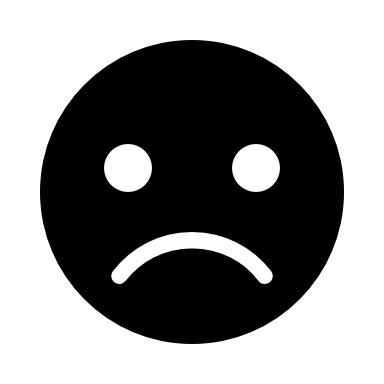


**SOME INFORMATION ABOUT YOU…**

We would like to ask a few more details about you to help us with our study.

| What is your relationship status? | Partnered (married/de facto)  Single  Other |
| --- | --- |
| How many people live with you at home? | ✍️______________ |
| What is the main language you speak at home? | Aboriginal/Torre Strait Islander language  English  Other |
| What is your employment situation? | Employed  Casual  Part-time  Full-time  Not working at the moment  Student  Retired/Pension  Home duties  Other |
| Has a doctor ever told you that you have one or more of these conditions? | Heart disease  High cholesterol  High blood pressure  Blood clot  Diabetes  Skin infection  Chest infection  Ear infection  Sexually transmitted infection  Asthma  Depression  Anxiety  Other mental health conditions  Drug or alcohol problem  Arthritis  Epilepsy  Thyroid problem  Stomach problem  Stroke  Lung cancer  Other cancer(s)  Other lung problems  Dementia  Hearing loss  Kidney problems  A disability  Other  ✍️ _____________________________  None of these conditions  Unsure |
| Which statement best describes your money situation? | I run out of money before payday  I have just enough money to get to the next payday  I have more than enough money to get to the next payday |

Thank you for completing the What Matters Survey!

**If you would like to go into the draw to win an Apple iPad, please enter your email address here:** ✍️**_____________________________________________**

**If you would like to be contacted to participate in a later stage of this study, please enter your email address here:**

✍️**_________________________________________**
